# Supplementary material for: Information Security Behavior in Health Information Systems: A Review of Research Trends and Antecedent Factors
Source: Healthcare (Basel). 2022 Dec 14;10(12):2531. doi: 10.3390/healthcare10122531 (PMC9777837; doi:10.3390/healthcare10122531)
Supplement: Supplementary file 1 [file healthcare-10-02531-s001.zip › Table S2 - Summary of selected studies.pdf]

Table S2. Summary of selected studies.

No.	Studies	Observed behavior	Dependent variables	Antecedents Factor	Samples	Organization Type	Study Location	Methodology
**1**	Johnston & Warkentin (2008)	Information privacy compliance (DSB)	Behavioral intention	Self-efficacy, perceived organizational support, organizational status, occupational type.	Clinical & non-clinical staff	Hospitals, private clinics, physical therapy facilities, mental healthcare facilities	U.S	Quantitative (survey)
**2**	Brady (2011)	Security regulation (HIPAA) compliance (DSB)	Security behavior	Management support, security awareness, security culture, computer self-efficacy, security effectiveness	Non-clinical staff	Academic medical center	U.S	Quantitative (survey)
**3**	Liginlal et.al (2012)	Security regulation (HIPAA) non-compliance (USB)	HIPAA Privacy rule non-compliance	Human error (slip/mistake), organizational factors (understaffing, high turnover, low morale, heavy workload, and work environment)	Privacy officer	Healthcare organization	U.S	Qualitative (interview)
**4**	Foth et.al (2012)	Security policy compliance (DSB)	Data protection compliance	Attitude, perceived severity, perceived probability, subjective norms, data protection level, perceived usefulness, perceived ease of use, demography as control variables (age, gender, occupational group, time of occupation, type of hospital)	administrative staff, nursing staff, physicians	Hospital	German	Quantitative (survey)
**5**	Mussa & Cohen (2013)	Security Protection (DSB)	Behavioral intention toward utilizing access control best practices	Perceived behavioral control, subjective norms, attitude, perceived severity, perceived susceptibility, cues to	Physician, Nurse, allied health	Academic medical center	U.S	Quantitative (survey)

No.	Studies	Observed behavior	Dependent variables	Antecedents Factor	Samples	Organization Type	Study Location	Methodology
				action, perceived benefits, security awareness, self-efficacy, controllability				
**6**	Box & Pottas (2013)	Security policy compliance (DSB)	Information security obedience	Attitude, emotions (loss, deterrence, challenge, and achievement)	Clinical staff	Hospital	South Africa	Qualitative (conceptual study)
**7**	Kuo et al. (2014)	Security Protection (DSB)	Information privacy-protective responses	Collection, unauthorized access, secondary use, error	Patient	Hospital	Taiwan	Quantitative (Survey)
**8**	Humaidi et al. (2014)	Security policy compliance (DSB)	User's compliance behavior towards ISPs (DSB)	Perceived susceptibility, perceived severity, perceived benefit, perceived barrier, cues to action, self-efficacy, perceived working experience	Doctors, nurses, pharmacists, radiologists, medical assistants, health administrators	Hospital	Malaysia	Quantitative (Survey)
**9**	Box & Pottas (2014)	Security policy compliance (DSB)	Security compliant behavior	Body of Knowledge, Attitude, Behavioral Intervention, Skill, Misuse deterrence, Compliance Promoting	Clinical & non-clinical staff	Healthcare organization	South Africa	Qualitative (conceptual study)
**10**	Humaidi & Balakrishnan (2015)	Security policy compliance (DSB)	HIS security policies compliance behavior	perceived severity, perceived susceptibility, perceived benefits, perceived barrier, self-efficacy, perceived trust, management support	Doctor, support staff, health administrators	Hospital	Malaysia	Quantitative (Survey)
**11**	Fernández-alemán et.al (2015)	Security best-practice compliance (DSB)	Security practices on security control	Number of years in the position, gender, educational level, professional category	Administrative assistant, porter, clinical laboratory technician,	Hospital	Spain	Quantitative (Survey)

No.	Studies	Observed behavior	Dependent variables	Antecedents Factor	Samples	Organization Type	Study Location	Methodology
				(multi-activity or mono-activity)	nursing assistant, nurse, doctor			
**12**	Foth (2016)	Data protection regulation compliance (DSB)	Intention to comply with data protection regulations	Subjective norms, attitude, perceived behavioral control, punishment severity, detection certainty	Nurse, doctor, administration, medical assistant, physical therapist, etc.)	Hospital	German	Quantitative (Survey)
**13**	Ma et.al (2016)	Privacy protection (DSB)	Behavioral intention	attitude, subjective norm, perceived behavioral control, perceived usefulness, perceived ease of protection, compatibility, peer influence, superior influence, self-efficacy, facilitating conditions	Nurse	Hospital	Taiwan	Quantitative (Survey)
**14**	Yang & Lee (2016)	Healthcare information protection (DSB & USB)	Induction control intention &<br>Self-defense intention	Severity, Vulnerability, Response-efficacy, Self-efficacy, Security awareness, Security system satisfaction	Doctor, nurse, medical administrator	Hospital	South Korea	Quantitative (Survey)
**15**	Samhan (2017)	Avoidance behavior to using the system in an insecure environment (DSB)	Avoidance behavior	Avoidance motivation, perceived susceptibility, perceived severity, perceived threat, safeguard cost, safeguard effectiveness, self-efficacy.	Physician, Nurse, Radiologist, Lab specialist	Hospital	U.S	Quantitative (Survey)
**16**	Park et.al. (2017)	Security violation (USB)	Intention to disclose patient's health information	Personal norms, self-control, Health information security awareness (General information security awareness, Health	Nursing student	Nursing school	Korea Selatan	Quantitative (Survey)

No.	Studies	Observed behavior	Dependent variables	Antecedents Factor	Samples	Organization Type	Study Location	Methodology
				information security regulation awareness, Punishment security awareness)				
**17**	Sher, Talley, Yang, et.al (2017)	Privacy policy compliance (DSB)	Behavioral intention	Fear arousal, response efficacy, self-efficacy, response cost, subjective norm, perceived vulnerability, perceived severity	IT staff	Hospital	Taiwan	Quantitative (Survey)
**18**	Sher, Talley, Cheng, et.al (2017)	Privacy protection (DSB)	Intention to protect EMR privacy	Perceived Susceptibility, Perceived Severity, Perceived benefit, Perceived barrier, Cues to action, Self-efficacy	IT staff	Hospital	Taiwan	Quantitative (Survey)
**19**	Park et al. (2018)	Security violation (USB)	Intention to disclose patient's health information	Medical assessment (patient medical status), self-efficacy, health information security awareness (General information security awareness, health information security regulation awareness, punishment severity awareness)	Nursing student	Nursing school	U.S	Quantitative (Survey)
**20**	Humaidi & Balakrishnan (2018)	Security compliance behavior (DSB)	User's compliance behavior	Self-efficacy, perceived trust, management support	Doctors, support staff, health administrator	Hospital	Malaysia	Quantitative (Survey)
**21**	Pathania & Rasool (2019)	Security compliance behavior (DSB)	Employee behavioral compliance	Organizational factors (Incentives, Nature of Work, Social Relations), Individual Factor (Self-efficacy, Attitude, Trust), Power Styles (Reward	HR expert, psychologist, hospital administrator	Hospital	India	Mixed method (Interview – survey)

No.	Studies	Observed behavior	Dependent variables	Antecedents Factor	Samples	Organization Type	Study Location	Methodology
				Power, Legitimate Power, Referent Power, Expert Power, Coercive Power)				
**22**	Kessler et.al (2020)	Compliance, participation, high-risk security behavior (DSB & USB)	Security behavior	Information Security Climate (Practices, Importance, laxness), Demography (Occupational category)	Certified nursing assistants, dentists, pharmacists, and physician assistants	Hospitals, physicians' offices, rehabilitation centers, and nursing homes	U.S	Quantitative (Survey)
**23**	Alexandrou & Chen (2019)	Securit control compliance (DSB)	Intention to comply with security controls	Perceived security risk, Security measure efficacy, self-efficacy, safeguard cost, Perceived susceptibility, Perceived Severity, and Regulatory concern.	Doctors & medical students; nurse & nursing students, medical technologist; healthcare administrators & IT staffs	Hospital and clinicis	U.S	Quantitative (Survey)
**24**	Altamimi et.al (2020)	Security policy non-compliance (USB)	Security non-compliance	Neutralization techniques, peer influence, superior influence	Medical interns, IT managers, & IT staff	Hospital	Saudi Arabia	Qualitative (interview)
**25**	Coventry et.al (2020)	Secure and insecure behavior (DSB & USB)	Insecure cybersecurity behavior	Perceived barrier of secure behavior, facilitator of insecure behavior	Doctors, nurses, administration staff, IT staff, finance staff, lab technicians, medical consultants, health workers	Hospital and public health center	Ireland, Italy, Greece	Qualitative (Focus group discussion)

No.	Studies	Observed behavior	Dependent variables	Antecedents Factor	Samples	Organization Type	Study Location	Methodology
**26**	Tazkarji (2020)	Security policy compliance (DSB)	Intention to comply	Habit, facilitating condition, punishment, rewards/costs, social factors, fear, patient-centeredness	Nurse	Hospital	U.S	Qualitative (conceptual study)
**27**	Sarkar et.al (2020)	Risk-taking and damaging behavior (USB)	Pseudo-compliance behavior, ISP violation behavior	ISP violation intention, perceived certainty of sanctions, perceived severity of sanctions, professional groups	Physicians, nurses, support staff	Hospital	U.S	Mixed method (Interview – survey – interview)
**28**	Alanazi et.al (2020)	Security compliance (DSB)	Information security compliance behavior	Legal/punishment, religion/moral, Personality traits, subjective norms, self-efficacy, cost of compliance, general information security, technology awareness, information security policy, demographic characteristics.	Nurse, administrators, pharmacists, physicians, other	Hospital	Saudi Arabia	Quantitative (survey)
**29**	Jalali et.al (2020)	Security policy compliance (DSB)	Behavior	Workload, perceived risk, intention to comply, attitudes, subjective norm, perceived behavioral control, collective felt trust, trust in technology (reliability and functionality), control variables (emails, age, position, education)	Clinical and non-clinical staff	Hospital	U.S	Quantitative (survey – experiment)
**30**	Fauzi et.al (2021)	Risky cybersecurity behavior (USB)	Information security practices	Perceived stress level	Manager, doctor, nurse, pharmacy staff, nutritionist, medical record staff, IT staff	Hospital	Indonesia	Quantitative (survey)

No.	Studies	Observed behavior	Dependent variables	Antecedents Factor	Samples	Organization Type	Study Location	Methodology
**31**	Kuo et.al (2021)	Security policy compliance (DSB)	Intention to comply with ISP	Punishment severity, punishment certainty, top management support, SETA programs, internal auditing effectiveness	Physicians, nurses, administrative staff, and other healthcare professionals (radiological technologists, pharmacists, etc.)	Hospital, medical center	Taiwan	Quantitative (survey)
**32**	Yeng et.al (2021)	Information security practices (DSB)	Healthcare information security metrics	Security practices (password management, email use, Internet use, social network site use, incident reporting, mobile computing, information handling), psychological traits, social factors, cultural factors, social demographics	Clinical staff	Hospital	Norway	Qualitative (systematic review)
**33**	Lee & Seomun (2021)	Securing patients' healthcare information (DSB)	HIS behavior	HIS intention, threat appraisal (intrinsic rewards, extrinsic rewards, severity, vulnerability), Coping appraisal (response efficacy, self-efficacy, response costs)	Nurse	Hospital	South Korea	Quantitative (survey)
**34**	Dong et.al (2021)	Security policy compliance (DSB)	Intention to comply with health ISP	Top management beliefs about information security issues, organization control of information security issues, attachment, commitment, involvement, personal norms, attitude	Nurse	Hospital	Malaysia	Quantitative (survey)

No.	Studies	Observed behavior	Dependent variables	Antecedents Factor	Samples	Organization Type	Study Location	Methodology
**35**	Kim et.al (2022)	Risk-taking behavior (USB)	Prosocial intention to disclose patient's health information	Disease severity, impact on others (family and patient), impact on the self situational empathy, responsibility to disclose, control variable (gender, age, academic year, rule awareness)	Nursing student	Healthcare organization	South Korea	Quantitative (survey – experiment)

DSB: desirable security behavior; USB: undesirable security behavior
